# Going to the South

**Published:** 1857-09

**Authors:** 


### GOING TO THE SOUTH.

MR. EDITOR:—I was glad to observe in a late number of your Journal, some suggestions to invalids respecting a winter's residence at the South. The season is now approaching when many of those who are afflicted with consumptive disease, or are threatened with its premonitory symptoms, will begin to dread the chill autumn winds, and prepare to emigrate to a warmer climate. I shall say little or nothing in this letter about the fallacy of the idea that such a change of climate will cure or tend to cure consumption. Having spent the whole of the past winter in Florida, I wish to give to your readers some intelligence respecting matters interesting to every invalid contemplating such a journey.

It is a very significant fact that there were not, during the past winter, as many invalids in Florida, *by more than one half,*

as there were the previous year. Why such a marked diminution? Some say that invalids were afraid of the Indians who infested the Everglades in the extreme south of the peninsula. Billy Bowlegs would think himself and his crew of some consequence if they should be informed of this. Others have thought that the political estrangement existing between the two sections of our country is almost enough to account for the fact. One can hardly repress a smile at such reasons as these. Undoubtedly multitudes of invalids have become convinced, both by medical advice and by the testimony of those who have tried the experiment, that "going south" is *noi* the sovereign panacea which it was once supposed to be.

Nevertheless, many will yet resort to this means in the hope of curing, or at least alleviating, consumptive disease. To such, the communication of a few facts respecting the expenses and mode of living in Florida may be useful. It is a fact well known to every observer, that the great majority of those who go to the South for health, are persons of scanty or moderate means. Many of them are ministers of the Gospel, who have worn themselves out on half pay, and are turned off by their ungrateful people, who borrow or beg as they find it most convenient. To most invalids, the expenses of a winter's residence in the South are a serious burden. *Sixty dollars a month* is the very lowest figure for a single person, and with incidentals and the few luxuries which are added, this amount will be considerably exceeded.

The invalid who goes to Florida, almost invariably deprives himself of many things absolutely indispensable to comfort. The hotels and boarding-houses are mostly supported by invalids, but on very few tables will be found some of the articles of diet most essential to persons of weakly constitution. If one endeavors to economize at all in the matter of board, he must put up with the poorest possible fare. I did not find a good cut of beef in the whole State, and a good joint of mutton is a rare dish. Venison and fowl are plenty, but even upon a vegetable diet one can hardly secure variety.

If the invalid wants a hair mattress, he must bring his own with him; for the luxury is not to be obtained in Florida. Whether the moss which is universally used there for beds is a suitable substitute, I am unable to say. I rarely slept in a room with plastered walls and ceiling. Men have little or nothing to do with masons in building their houses. For amusement, one finds almost nothing. The rivers, it is true, have fishes, and the woods have game; but few invalids that I met were rugged enough for such sport. One must seek his society among sick people; there is scarcely any other to be had. Once or twice a week is as often as one can get letters from home, under the most favorable circumstances.

I went to Florida in the fall of '56, intending to remain six months; but three months had not passed before I was worn out with the dull monotony of such a life, and often almost frantic for want of something—*anything*, to divert and occupy the mind.

Procuring a hard trotting horse about the first of March, I travelled over two months in the saddle, towards the North. Such a journey has its advantages for an invalid; but it has also its serious objections. In the first part of our route we camped out in the woods, and found a bed upon the ground more welcome than such lodgings as were afforded in wild and almost uninhabited pine barrens. Through a journey of a thousand miles, from Florida to Virginia, we stopped for the most part in log houses, with wide crevices as the only media of light and ventilation. In some respects the journey was beneficial; but if we could have persuaded ourselves to take half the amount of exercise at home, or in a colder latitude, we doubt not that we should have reaped double the advantage.

